# Perceptions and Barriers to Accessing Myopia Management in the UK

**DOI:** 10.3390/children11121490

**Published:** 2024-12-06

**Authors:** Stephanie Kearney, Sophie Coverdale, Cheralynn Saunders, Mhairi Day, Lindsay Rountree, Kathryn Webber, Edward A. H. Mallen, Neema Ghorbani-Mojarrad

**Affiliations:** 1 Department of Vision Sciences, Glasgow Caledonian University, Glasgow G4 0BA, UK; 2 School of Optometry and Vision Science, University of Bradford, Bradford BD7 1DP, UK; s.l.coverdale@bradford.ac.uk (S.C.); c.saunders@bradford.ac.uk (C.S.); l.c.rountree@bradford.ac.uk (L.R.); k.webber@bradford.ac.uk (K.W.); e.a.h.mallen@bradford.ac.uk (E.A.H.M.); n.ghorbanimojarrad@bradford.ac.uk (N.G.-M.); 3 Wolfson Centre for Applied Health Research, Bradford Royal Infirmary, Bradford BD9 6RJ, UK

**Keywords:** myopia, cost-effectiveness, quality of life, disease risk

## Abstract

Background/Objectives: Perceptions and barriers to myopia management (MM) in childhood have not been fully explored within some countries, including the UK, where there is minimal public health education on myopia. Methods: The aim of this mixed-methods study was to explore perceptions of myopia and MM interventions using focus groups and a survey to obtain qualitative and quantitative data. Topics included the understanding of myopia, perceptions of MM, considerations when initiating MM, lifestyle risk factors, and barriers to uptake of intervention. Results: Parent awareness and understanding of myopia and MM is limited. Many parents felt that they had not been provided with sufficient explicit advice about their child’s diagnosis or treatment. Despite this, parents were aware of some of the protective lifestyle behaviours which may slow myopia progression. The common belief was that myopia can affect a child’s quality of life. The most common reason that MM had been recommended to parents by practitioners was to reduce disease risk. Conclusions: The cost and lack of public awareness that MM interventions are available were the main barriers to the uptake of MM. There is a need to improve practitioner communication of myopia and its management and, subsequently, improve the standard of children’s eyecare.

## 1. Introduction

Myopia (short-sightedness) primarily occurs due to excessive axial elongation. This increase in eye length is the main contributor to pathologies such as myopic maculopathy, particularly in those with higher amounts of myopia [1]. The prevalence of myopia has increased across the globe [2], most notably in East Asia [3]. In response to this global health concern, various myopia management (MM) interventions have been developed to try and slow the progression of myopia in childhood, including specialised glasses, contact lenses, and atropine [4].

In addition to these optical and pharmacological interventions, some countries have implemented public health initiatives that focus on behavioural factors or lifestyle changes to manage the prevalence of myopia. Such examples include national strategies to reduce school pressures and increase physical exercise in China [5] and public health awareness posters to encourage children to go outside in Singapore [6]. Within the UK, there are no current government-led campaigns or government initiatives regarding myopia and its effects on the individual. This is likely due to the much lower prevalence of myopia in the UK than in other parts of the world; in Anyang City, China, the prevalence of myopia is up to 67% in primary school children [7], in comparison to 18.6% by 17 years of age in Northern Ireland, UK [8], and 29.4% by 12–13 years of age in Birmingham, England, UK [9]. In the UK, parents typically rely on their eyecare practitioner (ECP) for up-to-date information and education on their children’s eye health [10]. Due to variable uptake of interventions by ECPs across the UK, partially due to scepticism surrounding the quality and possible bias of current training opportunities [11], it is unclear what parents’ and children’s beliefs and motivations are regarding MM. This is of great importance, as parental motivation and choice of intervention are influenced by current knowledge and understanding [12].

Lifestyle factors, such as spending less time outdoors, influence the onset of myopia but have a lesser effect on its progression [4]. Lifestyle may also influence the success of an intervention, with some reports indicating that myopia progression can be exacerbated by less time spent outdoors despite the use of MM interventions [13,14]. Therefore, it is important to consider parental understanding of the risk factors for myopia to ensure that lifestyle modifications can be successfully implemented at home.

Various factors influence a parent’s motivation for undertaking MM, including the perceived severity of their child’s myopia and the child’s perceived future susceptibility to myopic progression [15]. A greater understanding of parental motivation and barriers to engagement is key, as this can influence the initiation of MM by ECPs [16]. Some studies have investigated whether artificial intelligence could be used in informing the public about myopia and MM, and it has demonstrated some potential for effectiveness; however, it cannot be fully relied on due to the prospect of misinterpretation and false information [17]. In the UK, ECPs believe that parents are unaware of the risk of associated pathologies yet express discomfort in discussing the potential future impact of myopia [11]. Instead, they believe parents are more concerned about dependency on spectacles and thicker spectacle lenses [11]. Amongst Irish parents, 46% considered myopia to be a health concern, but an equal number reported myopia to be an optical inconvenience [18]. ECPs in the UK have reported affordability to be a significant issue and barrier for parents considering an MM intervention [11].

Further exploration of barriers to the uptake of MM by parents and children is required through direct engagement with these groups to explore their perceptions in greater detail. This study aims to explore parents’ and children’s understanding of myopia risk factors, alongside their awareness of and motivations for initiating MM, to identify barriers to MM implementation and uptake.

## 2. Materials and Methods

This research employed a mixed-methods approach, utilising both focus groups and surveys to provide a holistic exploration of parents’ and children’s perceptions. The use of focus groups provides depth and context, whilst the survey allows for a wide reach of topics to be explored.

This research adhered to the tenets of the Declaration of Helsinki. Ethical approval was granted from the Biomedical, Natural, Physical and Health Science (BNPHS) Ethics panel at the University of Bradford (number E2122/00013) and from the Health and Life Sciences Ethics committee at Glasgow Caledonian University (HLS/LS/A23/028). A checklist for the consolidated criteria for reporting qualitative studies (COREQ) within the focus groups is provided in Table S1. Written informed consent was obtained from all parents.

### 2.1. Focus Groups

A series of focus groups were conducted between April and November 2022 by S.C., N.G.-M., L.R. and K.W. Parents with myopic children or children who were at increased risk of developing myopia (e.g., if one or both parents were myopic) from across the UK were invited to attend through online noticeboards, forum/social media posts, and in-person posters. Children were welcome to attend the discussions and were specifically invited to contribute their opinions during a part of the focus groups.

Three focus groups were held online, limited to a maximum of six parents in each group to facilitate in-depth discussions over 90 min [19,20]. Short presentations on myopia, MM interventions, and research methods were delivered to participants to educate them on the topic. Discussions were conducted both before and after the presentations. This deliberative approach promoted the collection of high-quality data and allowed participants to form informed opinions on the topic [21].

The facilitators were all UK-qualified optometrists who ensured that all participants had equal opportunity to share their views and contribute to the discussion. The facilitators followed a semi-structured topic guide (Table S2), informed by the current literature, to help stimulate conversation when and if needed.

The number of focus groups conducted was guided by continued appraisal, using ‘information power’, which incorporates aspects such as the participants’ knowledge and experience and the quality of dialogue achieved [22]. Data collection ceased once the researchers unanimously agreed that no new information was being obtained from the participants, and further focus groups were unlikely to lead to the identification of different themes [19,23].

### 2.2. Survey

A survey was designed in REDcap (Research Electronic Data capture tools, https://www.project-redcap.org/, accessed on 1 December 2023) [24,25] (Survey S1). The questions collected data on participant demographics, beliefs of myopia, protective behaviours against the progression of myopia, and barriers to engagement. The majority of the survey questions were closed questions. For all of the questions, parents could select a response or could opt not to choose an answer.

The survey was preliminarily tested amongst a pilot focus group of researchers and ECPs (*n* = 9) to evaluate aspects of the survey design, including reader interpretation and content of questions, the terminology used, and the duration of the survey.

The survey was circulated from February 2024 to May 2024 to parents with children who had attended an MM clinic appointment at any time and who had commenced or were about to commence an MM intervention across a range of optometry practises across central Scotland, UK. This was a separate group of parents and children from the focus groups. It is worth noting that there is currently no financial support available for myopia management across the UK, and this does not vary between the different nations of Scotland, N. Ireland, England, or Wales.

### 2.3. Data Analysis

#### 2.3.1. Focus Group

Recordings were anonymised and transcribed using a clean, verbatim approach (S.C.) [23]. They were then thematically analysed by two researchers independently (C.S., N.G.-M.) [23], who summarised sections of the transcripts and generated code labels, an example of which is provided in Table S3. Coded data were then organised into themes considering both the frequency and saliency of the points being made [23,26]. These were then harmonised into a singular thematic network, agreed upon by the two researchers. A third researcher aided in the harmonisation of the network and resolving any disagreement in themes (S.C.).

#### 2.3.2. Survey

The percentage of responses for each question was captured and reported. Parents self-reported the amount of myopia both they and their child had by choosing one of the following definitions: low (−0.50 to −2.99 D), moderate (−3.00 to −5.99 D), high (equal to or more than −6.00 D), or unsure.

The McNemar test was used to evaluate changes in parental beliefs and concerns about their child’s myopia following consultation with their optometrist. All statistical tests were performed using Stata 13.1 (StataCorp, College Station, TX, USA) using a statistical significance level of 5% throughout (*p* < 0.05).

## 3. Results

A total of sixteen parents participated in the focus groups, with children aged between 2 and 17 years. Of the parents, 10 out of 16 were myopic themselves, while 13 had at least one myopic child. Five children also took part in the discussions. Only two of the parents reported that their children were currently prescribed MM options.

For the survey, a total of 47 parents participated, the majority of whom had at least one child undergoing MM (89%). For those with more than one child undergoing intervention, the following results are related to their oldest child. Demographic data for both the focus group and survey participants can be found in Table S4.

### 3.1. Parental Concerns and Beliefs About Their Child’s Myopia

The focus groups revealed that parents described concern and feelings of anxiety about their child being diagnosed with myopia. Parents were not only worried about their child’s poor uncorrected vision and its effect on their quality of life but also the inconvenience of needing to wear optical correction. Few parents were aware of the increased risk of ocular co-morbidity with increasing myopia; after being presented with relevant information, parents expressed increased concern. Parents expressed worry about continued myopia progression and potential implications in the future, particularly fast myopic progression and a potentially high final refractive error.

*‘You think what kind of life is your child going to have and what are they going to be able to do and see?’* Parent 1 Focus Group 2.

### 3.2. Communication by ECPs

Focus groups found that parental and child awareness of myopia, myopia progression, and the availability of MM was generally poor. Some parents expressed feeling guilty for overlooking their child’s refractive error symptoms when they were first diagnosed. On diagnosis, many parents and children perceived communication from ECPs as lacking important information and empathy and were inconsistent between ECPs. Conversely, in parents who said they were given detailed information by their ECP, there were reports of feeling overwhelmed by the volume of information. Consequently, parents expressed frustration at having to look for other, possibly unreliable sources of information, elevating feelings of apprehension and uncertainty.

Both parents and children were unaware of the range of optical and non-optical MM interventions available and reported instances of ECPs having preconceived notions about which options may be favoured by them. Parents advised the main emphasis from ECPs during the discussion of optical interventions (when given) had been on cost, although they had not necessarily been made aware of the length of time of treatment that would be required and therefore the ongoing cost requirements of MM. Some parents perceived that ECPs were disinterested in paediatric eyecare due to the inconsistency of the information given post-diagnosis and the perceived lack of ECP availability/convenient appointment times for children. Parents also spoke of a lack of empathy for any concern felt at diagnosis for both parents and children.

*‘I would be happier if [ECPs] gave you a little bit more information or made you feel a bit better about things.’* Parent 4 Focus Group 1.

### 3.3. Primary Parental Concern

Survey responses indicated that the single primary concern parents had reported before speaking to their ECP was that myopia may affect their child’s quality of life (41%, *n* = 19). This was followed by the risk of visual impairment (20%, *n* = 9), disease risk (13%, *n* = 6), having thick glasses lenses (7%, *n* = 3), the effect on schoolwork (4%, *n* = 2), and sports ability (4%, *n* = 2). A total of 16% of parents (*n* = 7) stated they had no concerns. Quality of life was analysed to determine if this concern was shared across parents with myopia in comparison to those without myopia. A total of 13% (*n* = 6) of responses were from non-myopic parents, whilst 26% (*n* = 12) of responses were from cases where at least one parent was myopic. There was no significant difference between these groups (*p* = 0.16).

### 3.4. Change in Parent Beliefs After Speaking with an ECP

In the survey, parents could select all beliefs that they held about myopia prior to and after speaking with their ECP. The most common beliefs held by parents surrounding their child’s myopia prior to speaking to an ECP was that myopia may affect their quality of life (*n* = 29), followed by their child needing thicker glasses lenses (*n* = 24) (Table 1). A total of two parents knew nothing about myopia despite having attended previous appointments with their ECP. There was a significant decrease in the number of parents reporting that they believed their child would need thicker lenses after speaking with their ECP (*p* = 0.04) (Table 1).

### 3.5. Reasons for Undergoing MM

Within the survey, parents could indicate all the reasons why their ECP recommended MM. The most common reason selected was to reduce disease risk (33%, *n* = 15). This was followed by slowing progression (30%, *n* = 14), improving quality of life (28%, *n* = 13), reducing risk of visual impairment (26%, *n* = 12), reducing the need for a thick spectacle lens (7%, *n* = 3), improving the ability to play sports (11%, *n* = 5) or complete school activities (15%, *n* = 7), and ensuring the suitability of laser eye surgery in the future (4%, *n* = 2).

The main reason for parents pursuing intervention was the effect of myopia on quality of life (40%, *n* = 19). This was followed by the risk of eye disease (21%, *n* = 10), the risk of visual impairment (19%, *n* = 9), none of the stated reasons or no other reason specified (11%, *n* = 5), having thick lenses when older (6%, *n* = 3), and concerns over suitability for laser eye surgery in adulthood (2%, *n* = 1).

### 3.6. Parental Considerations and Perceptions of MM

When asked about MM within the focus groups, parents had mixed opinions on the different MM options. There was some degree of general dubiety about any intervention, such as why it was not deemed to be the first-line treatment option by all ECPs, and general hesitancy over relatively new ‘technology’. There was also scepticism over how worthwhile treatment would be, as efficacy is not certain and often does not fully prevent further progression. Parents who themselves had mild degrees of myopia and had experienced no negative impact were less motivated to pursue treatment for their children. Those who perceived their own myopia as undesirable, however, for example, those who had experienced myopia-related pathology or difficulties with vision, were more eager to pursue treatment for their children.

Most parents and children were open to or interested in using MM interventions, although the preference for intervention options varied. For some, a non-spectacle option such as contact lenses was valued, as this was seen as a way to prevent bullying, improve self-esteem, and enable the child to have spectacle-free periods of time. Some parents believed that orthokeratology would also potentially allow them some control and compliance monitoring at home. Others questioned the practicality of non-spectacle modalities, with uncertainty over whether their children were mature enough and would cooperate sufficiently with the required contact lens care regimen or eye drops. For some intervention options, there was hesitancy as to whether it would fit into the child’s lifestyle, such as difficulty with inserting and removing contact lenses or instilling drops. Parents and children alike raised concerns about the invasiveness and potential discomfort of some interventions, particularly with orthokeratology. There were general concerns over contact lens safety, particularly about the risk of infections and ‘losing the lens in the eye’. Parents noted such information had come from potentially untrustworthy online sources and acknowledged that clarification with ECPs had alleviated most concerns. Parental experience contributed heavily to the modality of choice for their children, such as being bullied at school for wearing spectacles or having experience using contact lenses themselves.

*‘I’d have a fear of them rolling into the back of my head… [I’ve heard] horror stories of people who slept in them. It’s kind of really put me off.’* Child 1 Focus Group 2

The majority of parents in the survey (82%, *n* = 27) reported that having myopia themselves increased the concern they have for their child’s myopia. The most common belief surrounding the aim of intervention was that it slowed myopia progression (91%, *n* = 43), with only a minority believing it stopped myopia progression (7%, *n* = 3), reversed myopia (2%, *n* = 1), or who were unsure (2%, *n* = 1).

### 3.7. Lifestyle Considerations

A common belief by parents within the focus groups was that an increased time spent on digital devices was a main causal factor for myopia. They discussed the perceived impact of schools in embedding digital technology into their children’s school lives and the lack of encouragement for outdoor activities at break times. Parents also discussed the prolonged use of digital devices outside of school, especially mobile phones which have become a large social aspect of children, teenagers, and young adults’ lives. Parents also described the impact of the COVID-19 lockdowns, with children spending even more time on screens for gaming, socialising, and education, and the potential ongoing impact this has had.

When considering lifestyle change suggestions, parents expressed issues about implementing more time outdoors. In the UK, they believed the lack of daylight hours and inappropriate weather conditions was an issue, while others discussed being cautious about letting their children outdoors unsupervised due to safety concerns, which are heightened compared to when they themselves were children. Parents also mentioned the feeling of increased struggle with getting their children to reduce their time using digital devices.

*‘There are lots of clubs at lunchtime—chess club, coding club… that’s not taking them outside.’* Parent 3 Focus Group 2.

When asked which behaviours may help in slowing the worsening of myopia, spending more time outside (94%, *n* = 44), taking frequent breaks from near work (94%, *n* = 44), and maintaining a working distance of at least 30 cm (83%, *n* = 39) were reported by the majority of parents responding to the survey. The majority of parents also believed that reducing the amount of time watching TV (70%, *n* = 33), wearing glasses full time (66%, *n* = 31), and ensuring room lights are on full when reading (57%, *n* = 27) were protective against myopia progression. A small number of parents reported that part-time wear (6%, *n* = 3) and eating fruit and vegetables (19%, *n* = 9) were protective behaviours.

### 3.8. Barriers to the Uptake of Intervention

Cost was the most commonly reported barrier to intervention in both the survey and focus groups. Table 2 provides a list of barriers and supporting quotes from the focus group discussions.

Parents resented having to pay additional costs for MM compared to traditional single-vision spectacle options and often viewed it as an optional extra rather than a necessity. Cost was a particular worry for parents with multiple children requiring treatment. The risk of the child losing or breaking a relatively expensive treatment was also a concern, creating additional costs. Some parents were distressed that they were unable to afford MM options for their child and expressed surprise and disappointment that MM is currently not fully subsidised by the NHS, as many expected all paediatric eyecare to be fully subsidised. Parents were also wary of costly MM options, with ECPs being perceived as profiteering and the eyecare sector, more broadly, being viewed as a commercial enterprise rather than a health care profession.

*‘I feel really bad sometimes that I can’t afford that.*’ Parent 4 Focus Group 3.

**Figure 1:**
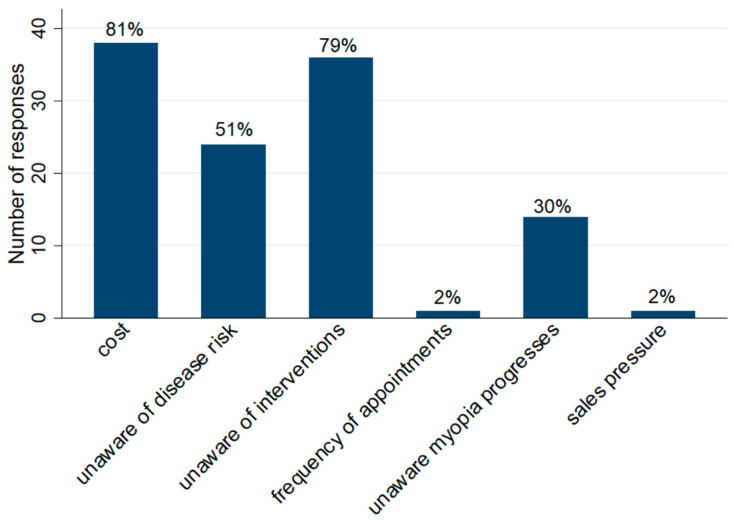
Bar chart depicting the number of responses relating to each barrier preventing the uptake of intervention by parents (survey data).

As mentioned, the survey results also indicated that the majority of parents reported that cost (81%, *n* = 38) was a barrier preventing the uptake of interventions. (Figure 1). Additionally, a lack of awareness that interventions are available was also reported by the majority of parents (79%, *n* = 37) (Figure 1), and was similarly identified in the focus groups.

Half of parents reported a lack of awareness of eye health risks of myopia (51%, *n* = 24) and a smaller proportion reported a lack of awareness that myopia can progress (30%, *n* = 14). Only one parent reported that the frequency of eye appointments and sales pressure may be barriers to the uptake of intervention. When asked how parents heard about interventions, 87% of parents reported that they heard from their optometrist and 13% from friends.

**Figure 2:**
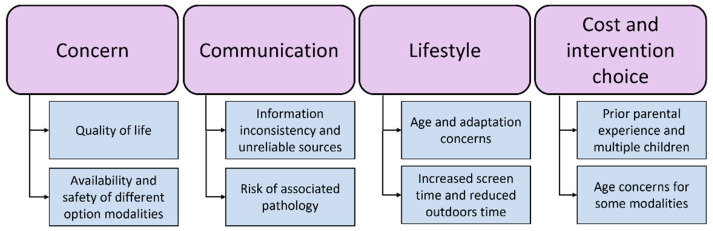
Summary of main themes (in larger purple boxes) and subthemes (in smaller blue boxes) identified via thematic analysis from the parent and children focus groups.

The main themes and the subthemes from the focus groups, as identified by thematic analysis, are summarised in Figure 2.

## 4. Discussion

To the authors’ knowledge, this is the first time that a mixed-methods approach has been used to obtain responses about myopia and its management directly from parents and children in the UK. This approach allows for both the depth of the issues to be considered using the focus group and the breadth of the issues to be explored using the survey. The primary barriers to the uptake of MM, as reported by parents, were the cost of intervention and lack of awareness that interventions are available. Cost remained a barrier despite the majority of parents within the survey group residing in more affluent areas. This suggests that accessing MM may be a challenge for all children, regardless of background. However, it is unclear if this relationship is present across other parts of the UK, as focus groups did not collect data on socioeconomic status. Further research is required to expand on this. Currently, across the UK, MM interventions are not publicly funded by the NHS. This may be partly due to the lack of clarity surrounding the cost-effectiveness of interventions and the burden of disease in the UK. Both of these metrics are key when determining national-level guidance and incorporation of any new health technology or medicinal product into the NHS by the National Institute for Clinical Excellence (NICE) [27]. Cost-effectiveness analysis of interventions to date has found atropine and spending more time outdoors to be the most cost-effective options in Hong Kong [28]; however, due to differences in health care delivery systems and product cost differences between countries, a UK-specific analysis is required.

In addition to the cost of an intervention, a lack of awareness that interventions are available, and that myopia may increase disease risk, were also barriers to MM by parents in this study. This may be due to variable uptake of interventions by ECPs within the UK due to a lack of confidence and knowledge surrounding topics such as clinical decision-making [11]. Indeed, there was no significant change in many parental beliefs about their child’s myopia after speaking to their ECP, which may suggest poor patient recall of information given by ECPs or further ECP training in MM is required. In fact, further detail on the delivery of information by ECPs on MM would be beneficial to understand this further, including whether the information is delivered in an appropriate language and in verbal and written forms, e.g., World Society of Paediatric Ophthalmology and Strabismus (WSPOS) [29].

Although there is perceived to be sufficient training available to ECPs, there is suspicion surrounding the quality of some of the content, with concerns about potential bias in training delivered by manufacturers [11]. Further sources of unbiased training from bodies such as universities may support ECPs when discussing this sensitive topic and help reduce concerns surrounding potential medical legal action [11]. An added complexity is that ECPs may avoid discussing intervention options with parents who they perceive may not be able to afford intervention [10]. This further highlights the requirement for exploration of the cost effectiveness of intervention. ECPs should, however, be careful to manage parental and child expectations with MM interventions. A recent report within a clinical population has found that there is considerable variation in the amount of slowing in axial elongation, with a standard deviation of 0.20 mm/year [30,31]. These variations in treatment effects should feature in discussions with parents, to allow them to consider the risk of non or low response versus the anticipated cost of intervention.

Another common barrier to interest in MM was a lack of awareness that myopia may increase the risk of associated eye disease. These findings have also been reported in previous focus groups with ECPs, where they reported avoiding discussion of disease risk to avoid concerning parents who could not afford MM [11]. A recent study has put into context the risk of developing myopia-related pathology and subsequent visual impairment by considering risk in absolute terms instead of relative terms [32]. Relative risk assesses the strength of the association between myopia and the disease without providing the raw risk profile, whereas absolute risk more appropriately contextualises the clinical significance of myopia as a risk factor for the child [32]. For example, in an East Asian child with low myopia, the absolute risk of visual impairment from retinal detachment, myopic maculopathy or glaucoma in adulthood increases to 7.89 in 100 if they develop high myopia. If stated in relative terms, this equates to an increase of 422% [32]. This allows for risk to be discussed in a less overwhelming manner. This, with the addition of the use of visual support aids such as pictographs and icons [32], should provide a more productive and balanced conversation for parents and children. In summary, absolute risk can contextualise the risk for that child as an individual rather than their current ’elevated’ relative risk [32].

A significant concern reported by parents was the effect myopia may have on their child’s quality of life. It is unclear which aspects of their child’s quality of life parents considered when completing the survey. It may be that parents are considering aspects of quality of life not influenced by disease or visual impairment, such as the social and emotional impact of having myopia in the absence of eye disease. High myopia is associated with a lower health-related quality of life [33], and this form of quality of life is the perceived effect of how disease and treatment may affect physical and psychological well-being, as well as social aspects of life [34]. Low and moderate amounts of myopia have been associated with lower vision-related quality of life [35], and this type of quality of life relates to visual function, including difficulties with vision-related daily tasks [35]. However, it should be noted that this research has been conducted in Asia where differences in culture with the UK may influence perceptions of quality of life. Whilst the majority of research has focused on the effects of myopia on disease risk, these risks are most notable for those with high myopia [36]. Therefore, it is important to consider how myopia, regardless of severity, may affect the quality of life relating to visual function and well-being.

Various aspects of parents’ beliefs and knowledge surrounding myopia were explored. There was no significant change in parents’ beliefs surrounding their child’s myopia after speaking with an ECP, with the exception that significantly fewer believed their child would have thicker glasses lenses. Having thicker lenses has also been reported as an observation from ECPs in the UK as a main concern of parents when discussing intervention [11]. The majority of parents also identified relevant risk factors for myopia progression, including near work habits, less time spent outdoors, and wearing MM intervention just part-time. However, parents also reported spending less time watching TV and having the room lights on full when reading as risk factors, despite having attended at least one appointment for MM. Evidence does not report an association between watching TV and myopia [37]. The causative role of dim room lighting in myopia requires further exploration [38,39]. This suggests that further parent education surrounding behavioural modifications may be, in part, required.

There are some limitations of this study requiring consideration. One limitation includes recall bias. However, to the author’s knowledge, this is the first time that responses have been collected using a mixed method approach, obtaining responses directly from parents and children in the UK. This approach allows for both the depth of the issues to be considered using the focus group and the breadth of the issues to be explored using the survey. Additionally, within the survey, for parents with more than one child undergoing intervention, this study only analysed responses pertaining to the oldest child. However, some parents may have had younger children who may have undergone intervention first, therefore influencing the beliefs they had surrounding their oldest child. This is unlikely to have significantly affected results as the majority of parents (89%) had only one child using the intervention.

## 5. Conclusions

The majority of parents stated that the greatest concern regarding their child’s myopia was the effect on quality of life. The majority of parents also correctly identified risk factors for myopia, although inaccuracies surrounding these risk factors indicate that further parent education would be beneficial. The cost of the interventions and lack of awareness that MM interventions exist are the primary barriers to parental uptake of interventions. There is a lack of awareness of the disease risk associated with myopia and the effectiveness of MM. Further research is required into the delivery of information by ECPs about MM to help overcome these barriers, and additional information on the cost-effectiveness of interventions is needed before state support can be considered. Whilst ECPs should consider quality of life and disease risk when discussing interventions with parents, unbiased sources of training may help support ECPs in discussing these sensitive topics.

## Figures and Tables

**Table 1:** The change in parental beliefs about their child’s myopia before versus after speaking to their eyecare professional (ECP). * denotes a significant difference. The shaded boxes indicated a majority of responses.

Beliefs	Before Speaking to Their ECP	After Speaking to Their ECP	
Disease risk	12 (25%)	18 (38%)	*p* = 0.15
Risk of visual impairment	18 (38%)	17 (36%)	*p* = 0.78
Effect on sports	20 (43%)	14 (30%)	*p* = 0.18
Effect on schoolwork	16 (34%)	11 (23%)	*p* = 0.18
Thick glasses lenses	25 (53%)	16 (34%)	*p* = 0.04 *
Suitability for corrective laser surgery in adulthood	9 (19%)	8 (17%)	*p* = 0.65
Quality of life	29 (62%)	27 (57%)	*p* = 0.79
Nothing known	2 (2%)	0	*p* = 0.38

**Table 2:** Table describing the barriers to uptake of intervention experienced by parents in the focus group (FG) accompanied by quotes from parents.

Barrier	Quote
Significant financial cost of interventions, including for multiple myopic children	*‘It’s really expensive. I keep thinking if they come down in price, great, but otherwise, it’s just not an option.’* Parent 4 FG3
Lack of full subsidisation by the National Health Service (NHS)	*‘I think it’s a massive, disturbing problem that should be given more prominence by the NHS.’* Parent 3 FG2
Perception of eyecare as a commercial enterprise	*‘We did start with an optician that bombarded us with some information without really giving us all the background to it, and it felt like they were really just financially wanting more money out of us for no good reason.’* Parent 1 FG3
Lack of parental awareness of the potential impact of myopia, the availability of myopia management interventions, and the importance of paediatric eyecare	*‘Yeah, I’m surprised that short-sightedness affects so many people. I didn’t realise it was that common.’* Parent 6 FG2
	*‘We’ve held back going for any lenses that are going to slow it down because we want to know, before we commit to [MM], what are the options out there?’* Parent 1 FG3
	*‘I think I did understand the importance of having a regular check-up, but I don’t think I realised that there was an increased risk of some of those conditions.’* Parent 4 FG3
Parental scepticism over new treatment options and technologies	*‘With them being so new, I suppose there’s not enough studies and research.’* Parent 3 FG3
Difficulties implementing lifestyle changes	*‘It’s very hard, especially with two years of COVID and lockdowns and being forced inside more or less, it’s really difficult. What have kids got to turn to—it’s either books or technology, isn’t it?’* Parent 4 FG1
Inconsistent communication and lack of guidance from ECPs	*‘We went to hospital for a diagnosis and weren’t really told much… You’ve just got Google to count on.’* Parent 2 FG1
Unreliable or untrustworthy information sources (online)	*‘[Information] is quite easy to come across, but it’s hard to know what to trust. ’Cause you don’t know which websites to trust and which not to trust.’* Parent 2 FG1
Practicality and safety concerns for some myopia management interventions	*‘He doesn’t want to put stuff in his eyes. He won’t want to put drops in.’* Parent 1 FG3
Perceived lack of maturity of children	*‘We completely ruled out hard contact lenses because we just felt she was too young.’* Parent 3 FG2
Poor local availability of MM intervention	*‘…but even they didn’t have myopia… the special lenses that we’ve been spoken to about.’* Parent 1 FG3
Accessibility of paediatric eyecareECP/practice disinterest in providing paediatric eyecare	*‘We found it hard to get the information and it be consistent across health carers and even just getting appointments.’* Parent 1 FG3
	*‘I didn’t get any feedback. I didn’t get any guidance, any support, anything, and I were taken aback ‘cause I were gutted that this had happened, and then I were just sent on my way with a prescription and that were it.’* Parent 2 FG1

## Data Availability

The data presented in this study are available upon reasonable request from the corresponding author. The data are not publicly available due to ethical reasons.

## Supplementary Materials

The following supporting information can be downloaded at https://www.mdpi.com/article/10.3390/children11121490/s1. Table S1. Consolidated criteria for reporting qualitative studies (COREQ): the 32-item checklist. Table S2. The semi-structured topic guide used by facilitators in focus groups to help stimulate conversation when and if needed. Table S3. Examples of code labels which were generated from transcribed recordings from focus groups to facilitate thematic analysis. Table S4. Demographics of participants in the focus groups and survey. Survey S1. Parent Motivations for Myopia Management Survey.
