# Supplementary material for: Perceptions and Barriers to Accessing Myopia Management in the UK
Source: Children (Basel). 2024 Dec 6;11(12):1490. doi: 10.3390/children11121490 (PMC11674830; doi:10.3390/children11121490)
Supplement: Supplementary file 1 [file children-11-01490-s001.zip › Survey S3.pdf]

Parent Motivations for Myopia Management Survey

This section will ask questions about you

What is your age in years?

What is your postcode? This is useful for us to know where in the country you live.

If you live outside of the UK, what country do you live in?

What is the highest level of education you have obtained?

- GCSES/O Levels/Standard
- Grade/National4/5 Scottish highers/A levels/AS Levels HND or HNC or equivalent Bachelor's degree
- Master's or doctoral degree
- Other (Please state)

What is your gender?

- Male
- Female
- Other (please state)
- I'd rather not say

What is your ethnicity?

- White Mixed/multiple ethnic groups
- Black/African/Caribbean/Black British
- Asian/Asian British (excluding Chinese)
- Chinese
- Other (please state)
- I'd rather not say

This section will ask about your children who are undergoing myopia management

How many children do you have who are undergoing myopia management?

- 1
- 2
- 3
- 4

- More than 4

What is your understanding of myopia management glasses or contact lenses?

- They reverse myopia
- They slow worsening of myopia
- They stop worsening of myopia
- I'm not sure
- Other (please state)

If you have more than 4 children undergoing myopia management, please consider your 4 youngest children undergoing myopia management for this questionnaire.

The following questions are in relation to your oldest child undergoing myopia management. If you have just one child undergoing myopia management please complete this section for them

How old in years is your oldest child undergoing myopia management?

What is the gender of your oldest child undergoing myopia management?

- Female
- Male
- Would rather not say
- Other (please state)

How much myopia does your oldest child undergoing myopia management have?
Consider the eye with the most amount of myopia (this will be the highest number). This is the strength of the sphere, or 'Sph' part of the glasses needed to correct myopia.

- -0.50 to -2.99
- -3.00 to -5.99
- Equal to or more than -6.00
- I'm not sure

As your child undergoes myopia management, you will have had appointments with an optometrist (commonly known as optician), the person who conducts the eye test.
We would like to find out about how much you understood about myopia in relation to this child before and after talking to an optometrist about them.

Which of the following did you believe about your oldest child's myopia before speaking to an optometrist? Please select all that apply

- Myopia could increase the risk of my child getting an eye disease

- Myopia could increase the risk of my child getting a visual impairment (sight impaired or blind) as a result of an eye disease
- Myopia could affect my child's ability to play sport
- Myopia could affect my child's ability to complete school work
- My child might have thick glasses lenses
- It could affect my child's eligibility for laser eye surgery when they're an adult
- Myopia could affect my child's quality of life
- Other (Please state)

Which one of the following was your main concern about your oldest child's myopia before speaking to an optometrist? Please select all that apply

- Myopia could increase the risk of my child getting an eye disease
- Myopia could increase the risk of my child getting a visual impairment as a result of an eye disease
- Myopia could affect my child's ability to play sport
- Myopia could affect my child's ability to complete school work
- My child might have thick glasses lenses
- It could affect my child's eligibility for laser eye surgery when they're an adult
- Myopia could affect my child's quality of life
- Other (Please state)
- I didn't have any concerns before speaking to an optometrist/optician

Why did your optometrist recommend myopia management for your oldest child? Please select all that apply

- To reduce the risk of eye diseases in later life
- To reduce the risk of visual impairment (sight impaired or blind) as a result of an eye disease
- To have thinner spectacle lenses in adulthood
- To improve my child's ability to play sport
- To improve my child's ability to complete school work
- To improve quality of life
- To improve my child's eligibility for laser eye surgery when they're an adult
- Other (please state)

Please now consider your understanding of myopia after speaking to an optometrist about this child's eyes.

Which of the following did you believe about your oldest child's myopia after speaking to an optometrist? Please select all that apply

- Myopia could increase the risk of my child an eye disease
- Myopia could increase the risk of my child getting a visual impairment (sight impaired or blind) as a result of an eye disease
- Myopia could affect my child's ability to play sport

- Myopia could affect my child's ability to complete school work
- My child might have thick glasses lenses
- It could affect my child's eligibility for laser eye surgery when they're an adult
- Myopia could affect my child's quality of life
- Other (Please state)

_

Which one of the following is the main reason for you to consider myopia management for your oldest child after speaking to an optometrist?

- Myopia could increase the risk of my child getting an eye disease
- Myopia could increase the risk of my child getting a visual impairment (sight impaired or blind) as a result of an eye disease
- Myopia could affect my child's ability to play sport
- Myopia could affect my child's ability to complete school work
- My child might have thick glasses lenses
- It could affect my child's eligibility for laser eye surgery when they're an adult
- Myopia could affect my child's quality of life
- Other (Please state)
- None of the above

The next question asks about family history of myopia

Do either of the child's parents have myopia?

- 1 parent is myopic
- Both parents are myopic
- None of the parents are myopic
- I'm not sure

How much myopia does the parent have?

- One parent is myopic by a small amount (-0.50 to -2.99)
- One parent is myopic by a moderate amount (-3.00 to -5.99)
- One parent is highly myopic (equal to or more than -6.00)
- I'm not sure

How much myopia does the mother have?

- They are myopic by a small amount (-0.50 to -2.99)
- They are myopic by a moderate amount (-3.00 to -5.99)
- They are highly myopic (equal to or more than -6.00)
- I'm not sure

How much myopia does the father have?

- They are myopic by a small amount (-0.50 to -2.99)
- They are myopic by a moderate amount (-3.00 to -5.99)
- They are highly myopic (equal to or more than -6.00)
- I'm not sure

Do the number of parents with myopia and the amount of myopia they have (as answered in the previous questions), increase your concern about your child's myopia?

- Yes
- No
- I'm not sure

The next section asks about some of your views on myopia management

Which of the following behaviours do you understand to be helpful in slowing the worsening of myopia? Select all that apply

- Spending more time outside
- Taking frequent breaks from near tasks
- Holding books and electronic devices at least 30cm away
- Reducing the amount of time spent watching television
- Having the room lights on full when reading
- Wearing glasses full time
- Only wearing glasses when watching the tv or looking at the board in school
- Eating more fruit and vegetables
- Other (Please state)

Where did you hear about myopia management glasses or contact lenses? (You may choose more than one option)

- Friends/Relatives
- Parents from school
- Optometrist/Optician
- Doctor
- Other health care provider
- Newspaper/Magazine
- Teacher
- Social media
- Other internet source
- Other (Please state)

In your opinion, which of the following prevents other parents from choosing myopia management for their child? Please select all that apply

- Cost of myopia management glasses or contact lenses
- Not being aware of the health risks of myopia
- Not being aware that myopia management glasses or contact lenses are available
- Having to attend more regular eye appointments
- Not being aware that myopia can worsen over time
- Feeling the optometrist/optician is trying t increase sales
- Other (Please state)
