# Supplementary material for: Perceptions and Barriers to Accessing Myopia Management in the UK
Source: Children (Basel). 2024 Dec 6;11(12):1490. doi: 10.3390/children11121490 (PMC11674830; doi:10.3390/children11121490)
Supplement: Supplementary file 1 [file children-11-01490-s001.zip › Table S2.pdf]

Table S2. The semi-structured topic guide used by facilitators in focus groups to help stimulate conversation when and if needed.

<table>
<tr><td>Discussion: Myopia and its implications</td><td>Is there anything you want us to clarify from the presentation?
What are your thoughts/feelings on myopia now since hearing the presentation?
Were you aware that there is a link between myopia and certain eye diseases? If yes, when were you made aware about this? If no, would you have wanted to be made aware?
What are your thoughts on the cost of myopia?
Any additional comments?</td></tr>
<tr><td>Discussion: Myopia management options</td><td>For children
Do you wear glasses and/or contact lenses?
What do you think about glasses/contact lenses/contacts that you sleep in?
If you could choose, which would you pick?
Would you be interested in taking part in an experiment to try out new lenses?
For parents
Is there anything you want us to clarify from the presentation?
Did you know anything about myopia management before this talk? If you knew about myopia management, when did you find out about this? If you hadn't, would you have liked to have known?
Would you opt for myopia management for your child compared to standard options to correct myopia? If no, what would be stopping you?
What do you think about these options for myopia management? Spectacles, contact lenses, orthokeratology, which would you pick?
If you found out from an eye test that your child didn't need glasses at the moment, but they were likely to become myopic/at risk of myopia, would you want to start a myopia management option at that point?
Any additional comments?</td></tr>
<tr><td>Discussion: PPI in myopia research</td><td>Is there anything you want us to clarify from the presentation?
If eligible, would you be interested in your child participating in research? Why or why not?
Is the language of the PIS suitable? (Ask participants if they have read this, provided in advance).</td></tr>
</table>

	• How do you feel about the timeline i.e. length of whole trial, number of visits, length of appointments?<br>• How do you feel about what is involved in the visits e.g. thoughts on use of eye drops in the study?<br>• How would you feel about your child being in the control group and not receiving treatment?<br>• Would you expect to receive any compensation for a research study? What compensation would you expect to receive? Would the level of compensation impact whether you would want your child to take part in the study?<br>• Any additional comments?
