# Supplementary material for: Perceptions and Barriers to Accessing Myopia Management in the UK
Source: Children (Basel). 2024 Dec 6;11(12):1490. doi: 10.3390/children11121490 (PMC11674830; doi:10.3390/children11121490)
Supplement: Supplementary file 1 [file children-11-01490-s001.zip › Table S3.pdf]

Table S3. Examples of code labels which were generated from transcribed recordings from focus groups to facilitate thematic analysis.

Data		Codes
Parent 4:	I would say it's a huge thing, the cost. I have glasses and normally buy two pairs, so I normally get a pair of glasses and then I'll get a pair of prescription sunglasses for driving. That costs me, and I don't get free eye test, so that cost me probably £20 for the eye test, last time I got both, it was about £150 for two pairs. My daughter has a pair of glasses, which I think we get, again, we get a voucher towards those, but she's at the age now at 17, where she wants contact lenses.	• Cost of myopia is a concern<br>• Cost of eye examinations is a concern<br>•Child has preference for contact lenses over spectacles
Parent 4:	I'm also paying for monthly contact lenses for her, which is in the region of about £36 for occasional use. I know she'd like to wear them every day, but it's not in our household budget for her to have, daily contact lenses, so it's a massive factor. It impacts on her self-esteem, and I feel really bad sometimes that I can't afford that. So the cost is massive. Actually when I took my son for his eye test and they said, "No, his vision is fine.", the relief was huge and it was a relief for him that he doesn't have to wear glasses, but it was also a big relief for me because I was thinking that would be another child, that would potentially glasses, contact lenses. It's huge. Massive.	• Unable to wear preferred correction (contact lenses) full time as too expensive<br>• Cost of contact lenses is a concern<br>• Spectacle wear having a negative impact on child's self-esteem<br>• Relief when second child didn't need spectacles due to expense
Parent 3:	Yeah, I've got similar thoughts as well. It's because I've been wearing contact lenses since I was 20 and glasses on top, so both of them add up and then if I've got to think that I've got three children if all three needed glasses and contact lenses. Then sometimes when they're doing sports and things they can't wear glasses, even if they want to so you've got to fork out for the contact lenses, which are not cheap at all.	• Parental contact lens wear<br>• High cost when using a combination of spectacles and contact lenses<br>• Contact lens correction for sports adds to the expense of myopia
Parent 4:	I've looked into contact lenses for myself, and I've got an astigmatism so the cost of contact lenses for me, I can't even go there. It's not even an option. It would be about £40/50 a month for me to get contact lenses. It's really expensive. I keep thinking, well, maybe if they come down in	• Parent not able to afford contact lenses due to high cost

	price, great, but otherwise it's just not an option.	
Parent 1:	I'm already… when the… when you talk about treatment or glasses and myopic lenses. I'm thinking about not just sunglasses for him now. I'm already on what lenses are out there to slow it down? Because that's our next viable option. Possibly with him being only seven. I mean the cost – we'll do whatever we need to, but yeah, the long-term commitment is unfortunate for any individual, isn't it? That that you just have to do what you can do financially and what fits into your daily budget and activities really. When we were talking about sports and things like that for him, we've already spoken about the head straps going on his glasses. We have damaged glasses regularly because he's boisterous, as any children will be they have their activities. It is something that just needs to be considered and it's yeah, it's hard.	• Parent wanting more information on possible myopia management options<br><br>• Implied: child's age will affect preference of treatment options<br><br>• Cost will affect preference of treatment options<br><br>• Child's lifestyle/hobbies will affect preference of treatment options<br><br>• Concern over damaged spectacles (cost implication)
