# Supplementary material for: Perceptions and Barriers to Accessing Myopia Management in the UK
Source: Children (Basel). 2024 Dec 6;11(12):1490. doi: 10.3390/children11121490 (PMC11674830; doi:10.3390/children11121490)
Supplement: Supplementary file 1 [file children-11-01490-s001.zip › Table S4.pdf]

Table S4. Demographics of participants in the focus groups and survey.

N/A indicates that these questions were not asked for this subgroup of participants.

*Several questions from the focus groups did not include explicit statements or answers from participants providing full self-reported demography. Therefore, this data is reported as missing. Proportions of the missing data for each question are listed in the table.

SIMD: Scottish Index of Multiple Deprivation. A national population-based tool, using postcodes. The SIMD combines 38 domains including income, education, housing and crime.

Demographic	Focus Group	Survey
Parent age	Mean 41.3 years old (SD ±8.5 years)<br>25% missing data *	Mean 44.9yrs (SD 5.3yrs)
Parent gender	83% female	85% female
Child's age	Mean 10.1 years old (SD ±4.0 years old)<br>38% missing data*	Mean 12.8yrs (SD 2.2yrs)
Child gender	60% female<br>13% missing data*	50% female
Parent ethnicity	75% White<br>13% Asian<br>6% Chinese<br>6% Other	76% White<br>17% Asian<br>7% Chinese
Parent education	N/A	28% Master's or Doctoral degree<br>49% Bachelor's degree<br>10% Higher National Certificate, Diploma or equivalent<br>13% school level qualification
Number of myopic parents	31% no myopic parents<br>19% one myopic parent<br>25% two myopic parents<br>25% unknown/unsure*	21% no myopic parents<br>40% one myopic parent<br>30% two myopic parents<br>9% unsure
Severity of parental myopia	N/A	24% low myopia<br>31% moderate myopia<br>38% high myopia<br>7% unsure

Severity of child's myopia	56% myopic<br>13% unknown/unsure*	13% low myopia
		30% moderate myopia
		33% high myopia
		24% unsure
Deprivation quintile	N/A	SIMD1 (most deprived) 11%
		SIMD2 11%
		SIMD3 13%
		SIMD4 27%
		SIMD5 (least deprived) 38%
